# Systems Mapping of the Complex Experiences of Family Caregivers and Care Recipients With Cognitive Impairment

**DOI:** 10.1093/geroni/igaf122.3849

**Published:** 2025-12-31

**Authors:** Doyoung Kim, Eleanor Hummel, Samuel Baxter, Anna Kahkoska, Leah Frerichs, Erin Kent, Katherine Ornstein, Kristen Hassmiller Lich

**Affiliations:** University of North Carolina at Chapel Hill, Chapel Hill, North Carolina, United States; University of North Carolina at Chapel Hill, Chapel Hill, North Carolina, United States; University of North Carolina at Chapel Hill, Chapel Hill, North Carolina, United States; University of North Carolina at Chapel Hill, Chapel Hill, North Carolina, United States; University of North Carolina at Chapel Hill, Chapel Hill, North Carolina, United States; University of North Carolina at Chapel Hill, Chapel Hill, North Carolina, United States; Johns Hopkins University, Baltimore, Maryland, United States; University of North Carolina at Chapel Hill, Chapel Hill, North Carolina, United States

## Abstract

Emotional well-being of family caregivers and older adult care recipients often fluctuates over the illness trajectory. Both parties influence each other’s emotional states within a complex web of interrelated factors, yet few studies have mapped the interconnected pathways underlying these dynamics. This study applied a participatory systems science approach to address this gap. Family caregivers of older adults (≥65 years) with dementia or other cognitive impairment residing in North Carolina participated in a four-hour group model building session involving drawing and explaining trend lines of emotional states and structured group discussions. Focus group data were analyzed using qualitative causal loop diagramming to depict factors, pathways, and feedback loops (i.e., a closed chain of causal connections) influencing emotional well-being over time. Between 12/2024 and 05/2025, ten sessions (n = 2–5 participants) were held with 29 unique caregivers (mean age 63.7 ± 11.9 years; 86.2% female; 72.4% White; 48.3% spousal; 62.1% caring for individuals at moderate-to-severe stages [FAST 5+]) of Alzheimer’s disease and other forms of cognitive impairment. Multiple complex system dynamics themes emerged, describing pathways and feedback loops that both influence and are influenced by changes in the emotional well-being of caregivers and care recipients. These themes relate to: caregiver and care recipient characteristics; interpersonal and family dynamics; social and formal support systems; healthcare and long-term care settings; legal and employment contexts; and technology use. Findings provide a systems-level foundation for designing interventions that holistically address interconnected drivers of well-being.

